# Optimizing US for HCC surveillance

**DOI:** 10.1007/s00261-024-04631-y

**Published:** 2024-11-25

**Authors:** Shuchi K. Rodgers, David T. Fetzer, James H. Seow, Kathryn McGillen, David P. Burrowes, Christopher Fung, Ashlesha S. Udare, Stephanie R. Wilson, Aya Kamaya

**Affiliations:** 1 Thomas Jefferson University, Philadelphia, USA; 2 The University of Texas Southwestern Medical Center, Dallas, USA; 3 Royal Perth Hospital, Perth, Australia; 4 Penn State Milton S. Hershey Medical Center, Hershey, USA; 5 University of Calgary, Calgary, Canada; 6 University of Alberta, Edmonton, Canada; 7 Stanford University, Stanford, USA

## Abstract

Ultrasound is the primary imaging modality used for surveillance of patients at risk for HCC. In 2017, the American College of Radiology Liver Imaging Reporting and Data Systems (ACR LI-RADS) introduced US LI-RADS to standardize the performance, interpretation, and reporting of US for HCC surveillance, with the algorithm recently updated as LI-RADS US Surveillance v2024. The American Association for the Study of Liver Diseases (AASLD) recommends reporting both the examination-level LI-RADS US Category as well as the US Visualization Score. The US Category conveys the overall findings of the exam and primarily determines follow up recommendations. The US Visualization Score conveys the expected sensitivity of the test and stratifies patients into appropriate surveillance pathways. One of the goals of routine surveillance is the detection of HCC at an early, potentially curable stage. Therefore, optimizing US technique is of critical importance. Increasing North American and worldwide utilization of LI-RADS US Surveillance, which includes technical recommendations, through education and outreach will undoubtedly benefit patients undergoing US HCC surveillance.

## Introduction

**Fig. 1:**
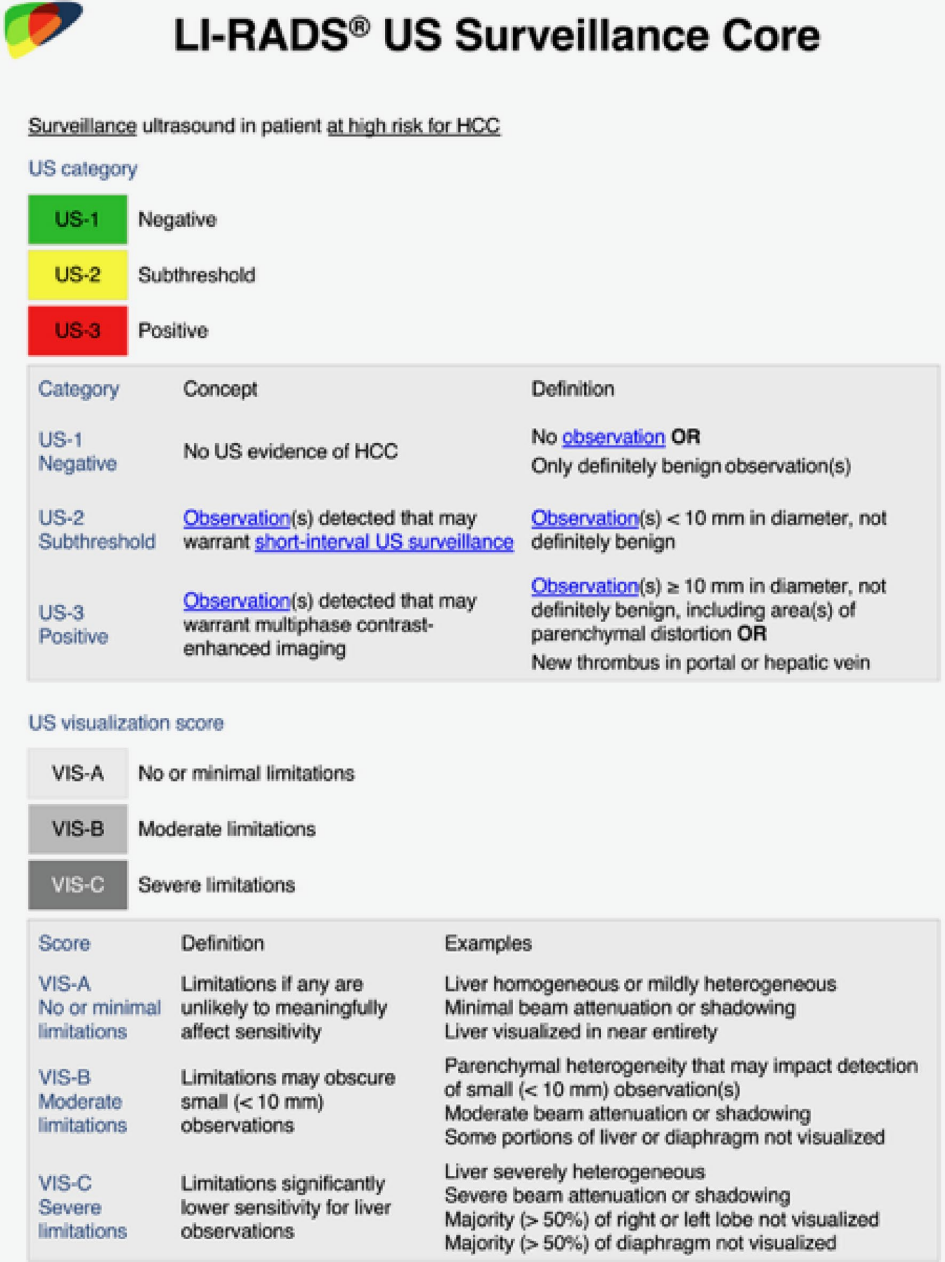
ACR LI-RADS US Surveillance v2024 Algorithm, reproduced with permission from ACR

**Fig. 2:**
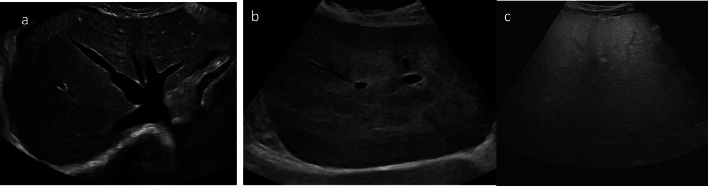
Visualization scores. **a** VIS-A, no limitations. Transverse grayscale US image in a 56-year-old patient with cardiac cirrhosis/congestive hepatopathy. **b** VIS-B, moderate limitations. Transverse grayscale US image in a 42-year-old patient with ethanol cirrhosis shows moderate parenchymal heterogeneity. **c** VIS-C, severe limitations. Transverse grayscale image in a 59-year-old patient with steatosis and metabolic dysfunction-associated steatohepatitis (MASH) cirrhosis shows poor penetration of the liver and complete obscuration of the diaphragm

Hepatocellular carcinoma (HCC) is the sixth leading cause of cancer and the third leading cause of cancer-related death worldwide [1]. Large cohort randomized control trials from China showed HCC-related mortality benefits in patients who underwent US-based surveillance [2, 3]. These data, and other smaller studies, have supported the use of US as the first-line modality for HCC screening and surveillance. The American Association for the Study of Liver Diseases (AASLD) continues to recommend US as the primary imaging modality combined with serum alpha-fetoprotein for semi-annual surveillance in a defined high-risk patient population [4]. ACR US LI-RADS v2017 provided a framework for performing, interpreting, and reporting US studies for HCC surveillance [5–7], and was recently updated as ACR LI-RADS US Surveillance v2024 [8]. Figure 1. The algorithm consists of an US Category (US-1: negative, no US evidence of HCC; US-2: subthreshold, an observation < 10 mm that is not definitely benign; and US-3: positive, an observation ≥ 10mm that is not definitely benign, or new portal or hepatic venous thrombus, that warrants further evaluation with a diagnostic contrast-enhanced US, CT, or MRI), and an US Visualization Score (VIS-A: no or minimal limitations, VIS-B: moderate limitations, and VIS-C: severe limitations) [5, 6] (Fig. 2). The 2023 AASLD Practice Guidance on prevention, diagnosis and treatment of HCC recommends reporting both the US Category and US Visualization Score to inform clinical decision-making and recall procedures [4]. A poor US Visualization Score (e.g., VIS-C) now provides a pathway to alternative screening strategies [9, 10]. The goals of this manuscript are to describe the challenges facing US surveillance, to provide feasible strategies to improve US image quality, to describe diagnostic imaging clues to prevent common interpretative pitfalls, and to promote adoption of LI-RADS US Surveillance into clinical practice.

### US sensitivity for HCC

Ultrasound has many advantages as a screening modality, including its wide availability, non-invasive nature, relatively low cost, and lack of radiation or need for contrast material [11–13]. However, multiple challenges may limit US image quality, including operator expertise, protocol variation among different sites, patient condition at the time of the scan, and steatosis or cirrhosis with parenchymal heterogeneity, leading to a lower visualization score [12, 14]. Fortunately, in many cases, visualization scores may improve on a follow-up US [10]. Etiologies for poor visualization scores are described in the next section.

The sensitivity of US for any stage HCC is reported as high as 84–94% which is acceptable for a surveillance exam [15, 16]. However, when focusing only on early-stage HCC, the sensitivity of US plus AFP was 63% in a recent meta-analysis [15]. Interestingly, this was comparable to CT for early-stage HCC detection which was found to be 62.5% in this same meta-analysis [13, 15]. Early-stage HCC is defined as a tumor burden meeting Milan criteria: a single lesion < 5 cm, or 3 lesions < 3 cm each, without vascular invasion or extrahepatic metastases [17]. A prospective study comparing the sensitivities of US and CT for detecting HCC in surveillance showed comparable values: 71.4% US sensitivity and 66.7% CT sensitivity, though a limitation is that US was performed every 6 months whereas CT was performed every 12 months [13]. Another study evaluating MRI and US performance for HCC detection showed an MRI sensitivity of 86% for HCC detection, which included early-stage disease [18]. While MRI outperforms US and CT, factors such as cost and accessibility make it impractical to use MRI for all patients eligible for HCC screening. In another study that evaluated how US Visualization Scores affect US sensitivity, VIS-A or VIS-B scored exams maintained a sensitivity for HCC of > 75%, while VIS-C scored exams had lower sensitivity at 27% [19]. Thus, poor visualization (VIS-C) exams have been shown to affect test performance, highlighting the importance of optimizing liver visualization in order to increase sensitivity and the need for alternative surveillance options for select patients.

The following section describes the challenges in US HCC surveillance and strategies to overcome them. Pitfalls and solutions for optimizing US HCC surveillance are summarized in Table 1.

**Table 1:** Summary of pitfalls and mitigating strategies for optimizing US HCC surveillance

Pitfall	Strategy
Infiltrative HCC	Recognize parenchymal distortion Look for venous thrombus Identify asymmetry between different segments or lobes of the liver Assess cine sweeps
Hepatic steatosis Patient with high BMI	Change to a lower frequency transducer Change to a penetration mode Image right lobe from multiple acoustic windows
Incomplete liver visualization HCC in liver periphery / sub diaphragm	Vary patient position Perform intercostal and subcostal scanning Encourage patient breathholding Capture cine sweeps Correlate with prior CT/MRI
US Operator and Radiologist Inexperience	Provide in-services and regular quality assurance meetings to review cases and equipment Use a dedicated, standard liver US protocol Increase operator/radiologist volume Utilize peer learning Identify Ultrasound specialists Engage “Super” techs to perform quality control of studies

### Challenge # 1: visualization C exam

Between 5 and 20% of patients may have inadequate visualization of the liver parenchyma (e.g., VIS-C exam), with the highest risk in obese patients with a body mass index exceeding 35 kg/m^2^, Child-Turcotte-Pugh (CTP) Class B or C cirrhosis, or cirrhosis secondary to alcohol or metabolic dysfunction-associated steatohepatitis (MASH) [9, 20, 21].

**Fig. 3:**
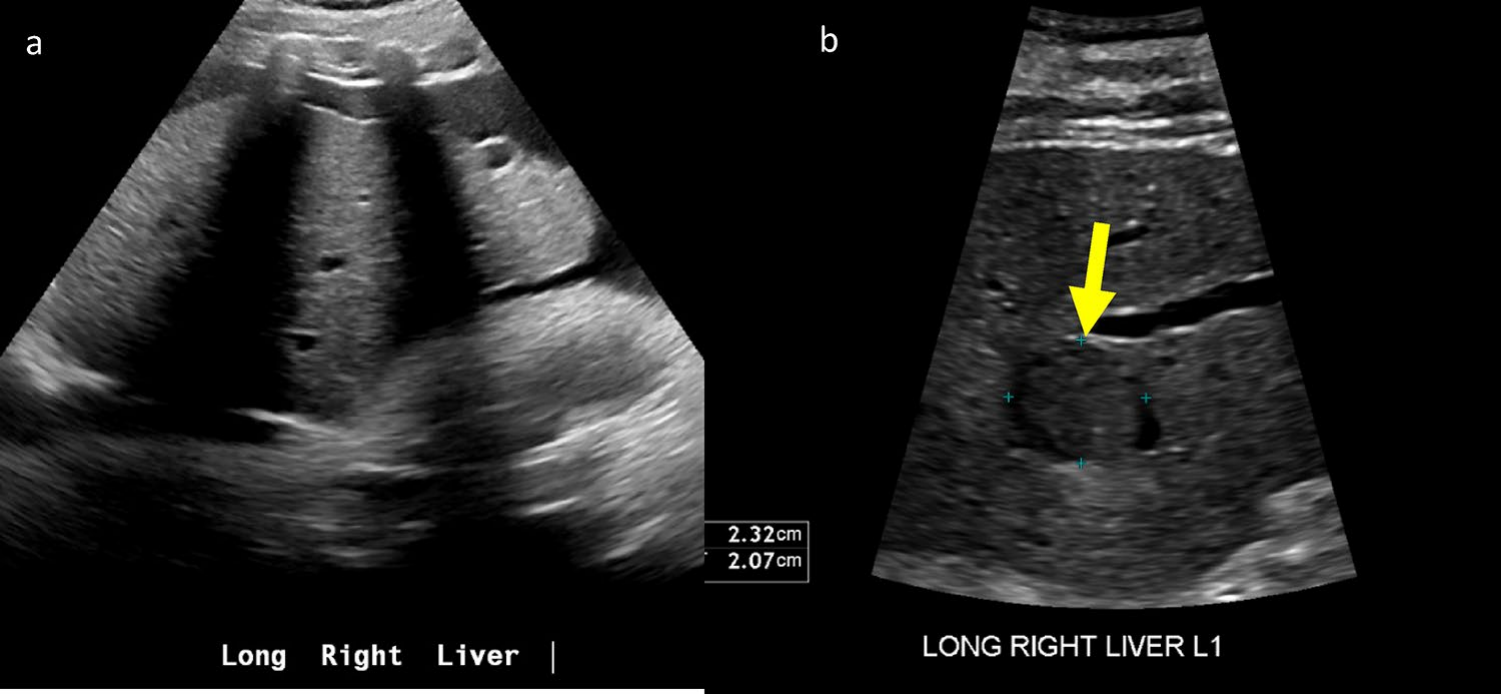
Visualization score improves in outpatient setting in a 60-year-old male with alcoholic cirrhosis. **a** Sagittal grayscale US image of the liver shows rib shadows and ascites, with no suspicious observation noted. The patient was hospitalized for decompensated cirrhosis and was unable to change positions or suspend respiration. **b** Sagittal grayscale US image of the liver performed a few months later in the outpatient setting shows a 2.3 cm hypoechoic solid nodule (arrow and in calipers), categorized as US-3 positive. This was characterized as LR-5 on subsequent MRI (not shown)

Cirrhosis with parenchymal heterogeneity due to nodules, fibrosis, and/or atrophy may make it exceedingly difficult to distinguish underlying lesions that would otherwise be suspicious for HCC [14]. Furthermore, severe steatosis may reduce sound penetration and conceal observations. Liver visibility may also be limited by the position of the liver, patient obesity, and overlying ribs and bowel. Additionally, optimal liver sonography requires patient cooperation to suspend respiration and change position, which are more likely to be compromised in in-patient or acute care settings. Figure 3. Therefore, ACR LI-RADS US Surveillance v2024 recommends deferring HCC screening to the outpatient setting whenever possible, acknowledging that patient needs and provider preferences may outweigh the drawback of decreased test sensitivity.

**Fig. 4:**
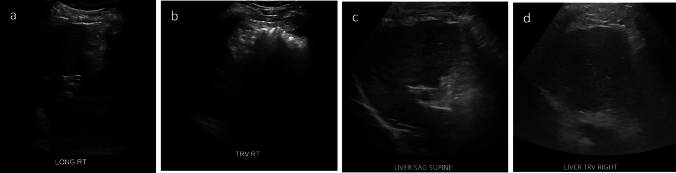
Change in visualization score on follow-up in a 33-year-old male with metabolic dysfunction-associated steatohepatitis (MASH) versus alcoholic cirrhosis. a and b. Sagittal and transverse grayscale US images show minimal liver parenchyma due to rib shadows in a and bowel gas in b. Follow up grayscale US 3 months later shows no limitations and adequate visualization of the liver. Over 50% of patients with VIS—C scores improve on follow up 10

For patients with a VIS-C scored examination, ACR LI-RADS US Surveillance v2024 now provides a new management pathway. Options include: repeating US within 3 months (which may apply to patients who have a chance of improving liver visualization on follow up) (Fig. 4), or proceeding directly to an alternative surveillance strategy if the patient has high risk factors for VIS-C. If a patient has two consecutive VIS-C scores, the likelihood of future examinations improving is significantly decreased. Therefore, alternative surveillance imaging strategies should be considered in these patients.

### Challenge #2: difficulty in detecting HCC due to small size, isoechogenicity, or an infiltrative pattern

HCC characteristics that may limit their sonographic visibility include small size, isoechogenicity to the liver, a subdiaphragmatic or peripheral location (“blind spots”), or an infiltrative growth pattern. “Blind spots” may include HCC in the hepatic dome, caudate lobe, regions adjacent to the inferior vena cava, subcostal liver, and the far lateral segment of the left hepatic lobe. This section will focus on interpretive patterns and sonographic techniques to improve US sensitivity for these types of HCC presentations.

**Fig. 5:**
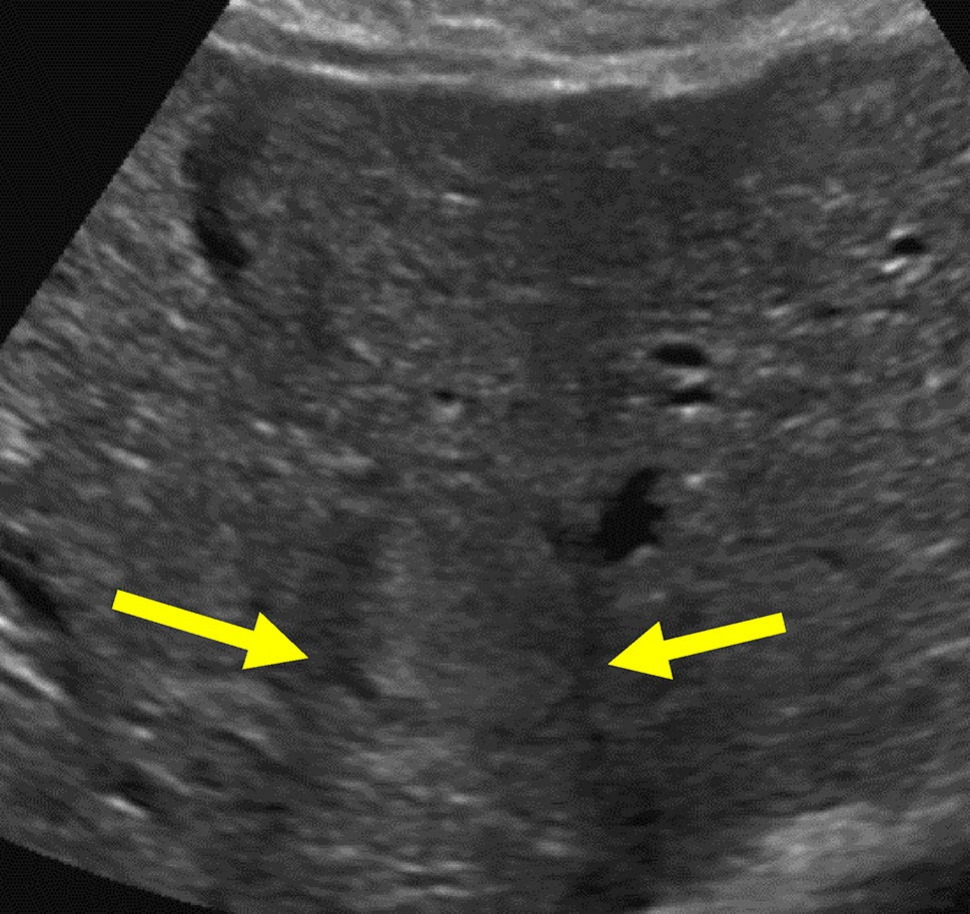
Isoechoic HCC. Transverse grayscale US image in a 58-year-old patient with hepatitis C cirrhosis shows an isoechoic HCC. The hypoechoic halo and refractive edge shadows (arrows) around the margins of the observation make it stand out against the background of the cirrhotic liver

While most HCC are hypoechoic to the liver or mixed in echogenicity, some may be isoechoic or hyperechoic, or the echogenicity may change as the tumor progresses [22]. When isoechoic or small, HCC may be indistinguishable from regenerative nodules or the background liver parenchyma [23]. Visualization may be improved by switching transducers or preset settings, decreasing dynamic range, and focusing on any subtle areas of heterogeneity, distortion, or abnormal color Doppler flow. Isoechoic liver lesions may also be appreciated by the refractive edge shadows that can emanate from the edges of the lesion Fig. 5.

A study that performed targeted prospective sonography of small HCCs detected by CT/MRI confirmed that small HCC in close proximity to the diaphragm are more difficult to appreciate on US [24] due to its inherently deep location, partially surrounded by lung, often requiring subcostal and intercostal sonographic approaches for visualization. The lateral segment of the left lobe of the liver, particularly after it crosses the midline and abuts the spleen, can also be an overlooked region of the liver. The LI-RADS US Surveillance liver protocol specifies to include the right and left liver edges to ensure complete liver visualization.

**Fig. 6.**
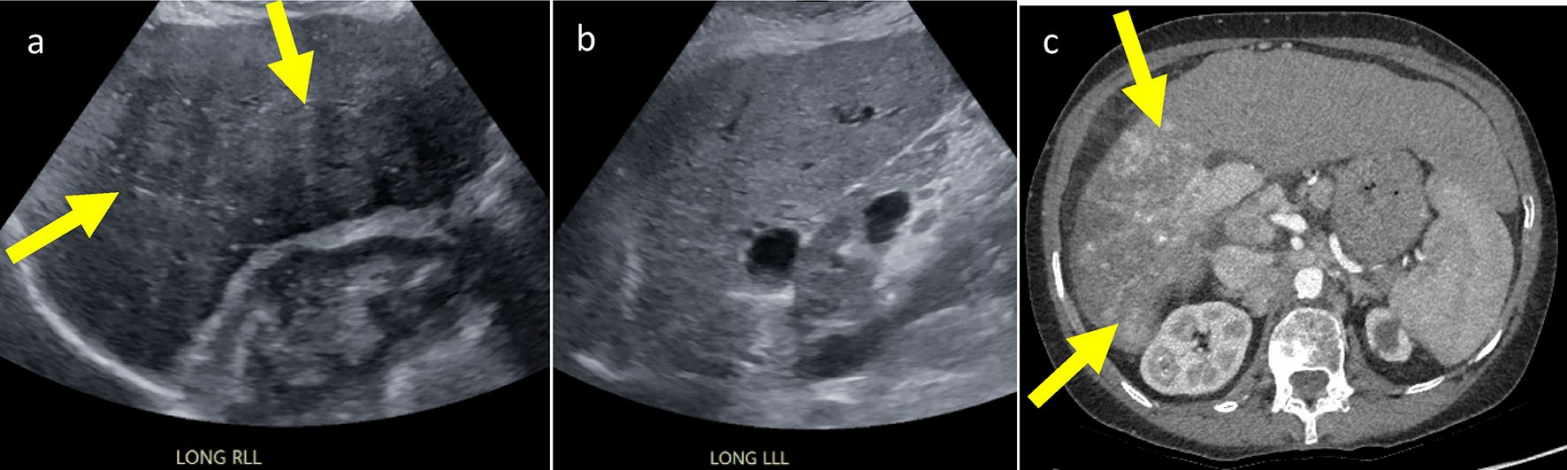
55-year-old female with ethanol cirrhosis and a 15 cm right lobe infiltrative cholangiocarcinoma not originally appreciated on US. Note that an infiltrative HCC could have a similar appearance. **a** Sagittal US image of the right lobe of the liver shows a large area of parenchymal distortion (arrows) with areas of refractive shadowing. **b** Sagittal US image of the left lobe of the liver, included for comparison, shows cirrhosis without parenchymal distortion. **c** Axial contrast enhanced arterial phase CT shows large area of heterogeneous enhancement (arrows) in the right lobe of the liver, corresponding to biopsy proven infiltrative cholangiocarcinoma. The right portal vein was not discretely visualized, presumably due to tumor in vein (not shown)

**Fig. 7:**
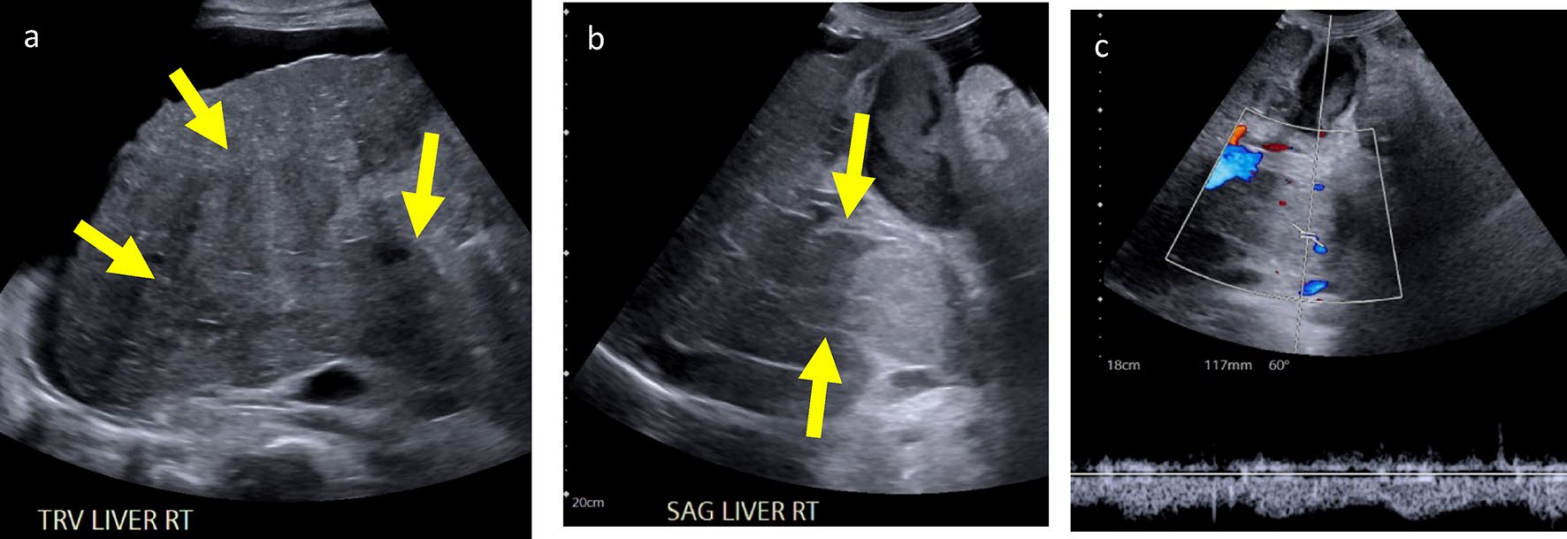
Infiltrative HCC and tumor in vein in a 62-year-old male with acute liver failure, presenting for urgent liver transplant evaluation. **a** Transverse grayscale US image shows a large area of parenchymal distortion in the right lobe of the liver (arrows), initially attributed to cirrhosis. The interpreting radiologist recognized the lack of a normal portal vein and requested additional images. **b** Sagittal grayscale US image of the porta hepatis using a larger field of view and the gallbladder (G) (filled with sludge) as a window, shows an expanded, thrombosed portal vein (arrows) with echogenic walls. **c**. Spectral Doppler image of the portal vein shows low resistance arterial flow diagnostic of tumor in vein

Infiltrative HCC comprises 7–20% of all HCC [25–27]. Its intrinsically poorly defined borders and marked heterogeneity can make it difficult to differentiate from typical features of cirrhosis. Somewhat counterintuitively, the large size of infiltrative HCC can make it difficult to identify on static US images, as they limit the comparison with unaffected background parenchyma [28]. However, there are some lesser-known features that can help diagnose an infiltrative HCC. One clue is parenchymal distortion, manifesting as an area distinctive from the background liver, often with refractive shadowing that results from differences in tissue composition and orientation; a feature which also corresponds to an US-3 positive finding. Figure 6. Small satellite lesions are also reported in approximately half the cases of infiltrative HCC and may be more readily identifiable. Additionally, infiltrative HCC is associated with tumor-in-vein in 68–100% of cases, making identification of a new venous filling defect an extremely useful diagnostic clue [25, 28] Fig. 7. The thrombus typically expands the vessel and most commonly involves the portal vein and/or its branches, though occasionally involves the hepatic veins and IVC [29]. The sensitivity for detecting tumor in vein based on arterial spectral Doppler waveforms in the thrombus is 62% with a specificity of 95%. Due to this, it should be noted that all new venous thrombus (even if not obviously tumor in vein), is considered an US-3 positive finding [28, 30].

### Challenge #3: protocol heterogeneity

**Fig. 8:**
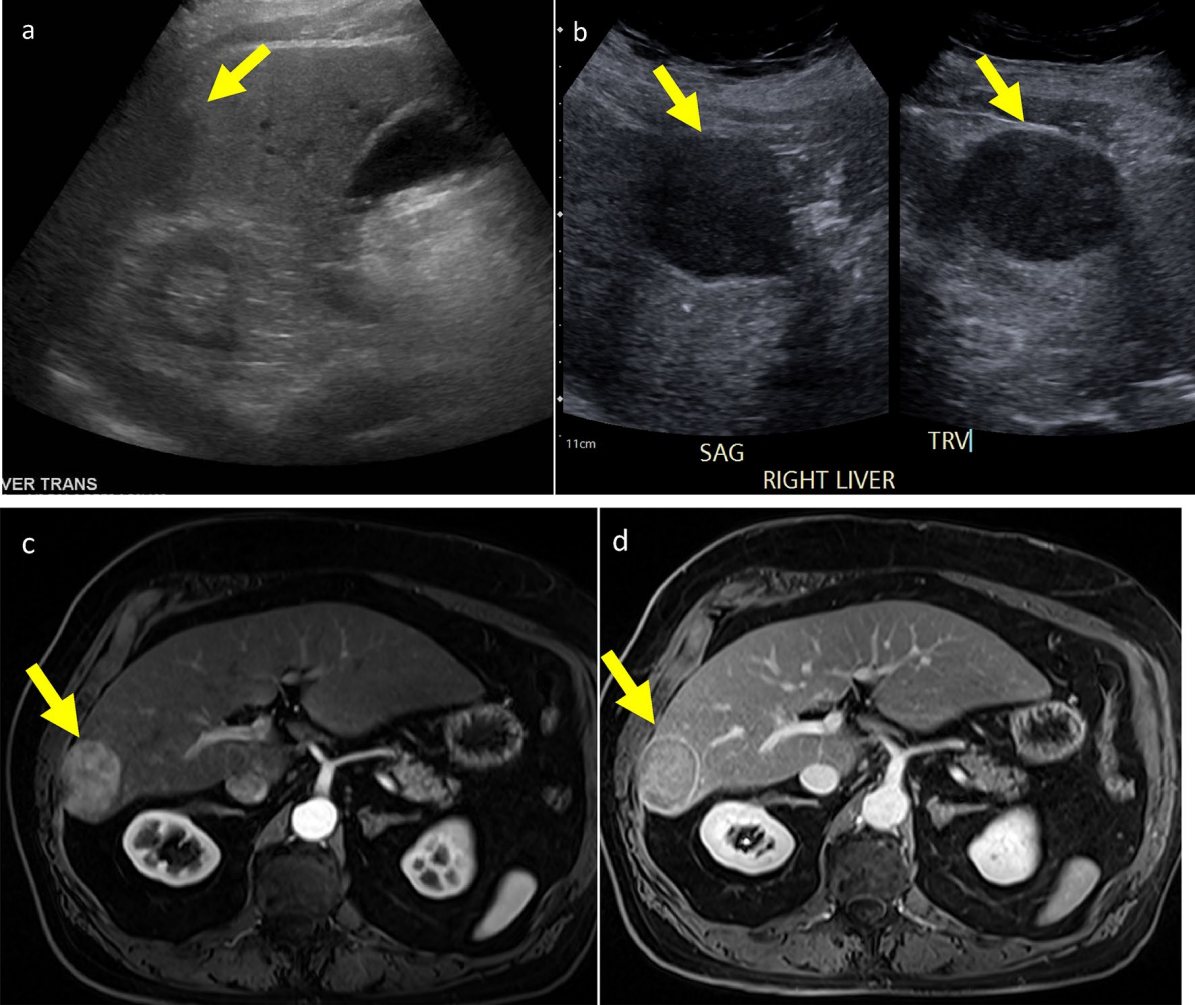
Liver mass initially not seen on routine abdominal US performed in a 63-year-old male with metabolic dysfunction-associated steatohepatitis (MASH) and F3 fibrosis. **a** Transverse grayscale US image right lobe of the liver shows a peripheral hypoechoic region (arrows), only visualized on retrospective review of the images, that may correspond to the observation detected in **b**. **b** Patient returned for US elastography 3 weeks later, and sonographer placed patient in a decubitus position. Sagittal and transverse grayscale US dual screen image of the liver shows a 4.6 cm solid hypoechoic mass (arrows). This case underscores the need to examine the liver’s periphery, which can be enhanced by placing the patient in a decubitus position, suspending respiration, and using intercostal and subcostal scanning. **c** and **d** Axial contrast-enhanced MRI of the abdomen in the arterial (**c**) and delayed (**d**) phases show arterial phase hyperenhancement of the observation with ‘washout’ and enhancing ‘capsule’, representing HCC

LI-RADS US Surveillance v2024 recommends the implementation of a dedicated liver surveillance imaging protocol that documents all portions of the liver with an emphasis on complete visualization of the liver. Standardized imaging protocols ensure examination consistency and reduce inter and intra-user variability. One single-center study compared HCC detection rates between a focused liver imaging protocol (as per US LI-RADS recommendations) to a general complete abdominal protocol. The authors found that between the two groups, there was a significantly higher detection rate of HCC among those who performed the focused protocol. The two groups had similar operator experience, patient demographics, and liver disease status apart from the focused group receiving 4 h of training in the LI-RADS protocol [31]. Figure 8. In addition, a dedicated liver protocol should include additional components important to the liver disease status, such as presence of bile duct dilatation, high-frequency linear transducer images of the liver capsule and underlying parenchyma [32], spleen length and volume, as well as evaluation for ascites. Complete imaging of other structures such as the kidneys, aorta, or pancreas is not always necessary in an HCC surveillance exam.

### Challenge #4: unfamiliarity with ultrasound equipment

Hardware improvements and software enhancements in newer imaging devices can significantly improve US grayscale and Doppler imaging, particularly in patients with obesity or severe steatosis. US operators are encouraged to gain the experience, knowledge and freedom to perform image and patient-specific scanner setting changes to optimize image quality. Image quality improves when the sonographer chooses the transducer (probe) or transducer center frequency most appropriate for the patient and their liver. Additional scanner settings may alter or enhance the echotexture of the liver, and the conspicuity of focal liver lesions. Detailed suggestions are included in Table 2.

**Table 2:** Optimizing US machine settings

Setting	Options	Outcome
Transducer (Probe)	Lower frequency curved (1–5 MHz) High frequency curved (2–9 MHz) High frequency linear (3-18 MHz)	Lower frequency curvilinear transducers are the workhorse for most patients, though are most ideal for those with obesity or steatotic liver disease Higher frequency curvilinear transducers preferred in pediatrics and small-to-average sized adults to achieve higher spatial resolution, as penetration is less of a concern Linear transducers are often helpful in evaluating the anterior liver capsule for nodularity, and underlying parenchyma for coarsening, signs of advanced chronic liver disease
Transducer frequency	Low frequency (Penetration Mode) Mid frequency (General Mode) High frequency (Resolution Mode)	Most contemporary transducers can operate at various center frequencies. Most systems have a Mid or General setting, which drives the transducer in the middle of its range (eg. for 1–5 MHz, a General mode would likely operate at ~ 3 MHz) Changing to a lower frequency (eg. 1 MHz), generally designated as Low or Penetration mode, allows for improved penetration. This is particularly helpful in patients with obesity or steatosis but comes at the expense of spatial resolution By changing to high frequency (eg. ≥ 5 MHz), spatial resolution is optimized but at the expense of penetration—this trade off may be acceptable in patients without significant steatosis
Harmonics	Off or On	Harmonics reduces image clutter and some artifacts in order to improve image clarity; however, this often comes at the expense of penetration For most modern systems and transducers, harmonics is active by default. In some cases, turning off harmonics may improve penetration but this comes at the expense of image clarity
Compounding	Off or On	Compounding reduces image clutter and some artifacts to improve image clarity, however at the expense of frame rate For most modern systems and transducers, compounding is active by default. Turning off compounding may improve frame rate, but at the expense of image clarity Compounding is automatically deactivated when using color or spectral Doppler, or when performing a contrast-enhanced US exam
Speckle Reduction	Various	Most modern systems leverage advanced speckle reduction techniques. These algorithms reduce image noise, often producing a smoother-appearing image. Contemporary techniques also provide edge enhancement and local image contrast improvements Many scanner presets are designed for specific tasks, such as liver or renal imaging, musculoskeletal, or vascular imaging. Many manufacturer-specific image clarify settings and tools are available and should be tailored for a group’s desired liver appearance with the goal of optimizing the detection and delineation of subtle liver lesions, particularly when isoechoic to background liver

### Challenge #5: adequate US operator and radiologist training

Ultrasound is subject to operator dependence and greater inter-user variability than other modalities such as CT and MRI. Although the experience and skill of the US operator, and proficiency and experience of the radiologist may impact the quality of the imaging examination and interpretation respectively, there is a paucity of literature on this topic. A recent study of over 10,000 surveillance liver examinations performed at two separate hospitals identified multiple factors that impacted visualization scores [33]. Not surprisingly, studies performed by more experienced sonographers and interpreted by subspecialized radiologists were associated with better visualization scores. However, sonographer and radiologist training on optimizing imaging technique can improve visualization scores and detection of HCC. These activities can range from sharing challenging cases, journal club presentations, peer learning conferences, or other quality assurance programs. One study evaluated the impact of on-site education on US-based HCC surveillance examination quality [34]. The authors performed quality assessment of examinations and found a significant decrease in examination failure and improvement in mean quality scores immediately after on-site education, though this drifted towards pre-education quality one year after, suggesting that continuous and recurring education is needed to maintain optimal surveillance efficacy.

### Challenge #6: resistance to cine sweeps

The ACR LI-RADS US Surveillance v2024 recommends a dedicated liver US protocol, with the suggested views listed in the core document online [8]. Multiframe cinematic clips (“cine sweeps”), defined as a recording of the dynamic imaging as the US operator pans through a selected field of view, are now recommended. The LI-RADS US Surveillance v2024 Core suggests at least 3 simple yet specific cine sweeps—(1) transverse subcostal large field of view sweep, (2) transverse right lobe, and (3) sagittal left lobe. Additional cine sweeps can be obtained as per institutional protocol. If a focal observation ≥ 1 cm is identified, focused sweeps through the observation in two planes are also recommended [8].

**Fig. 9:**
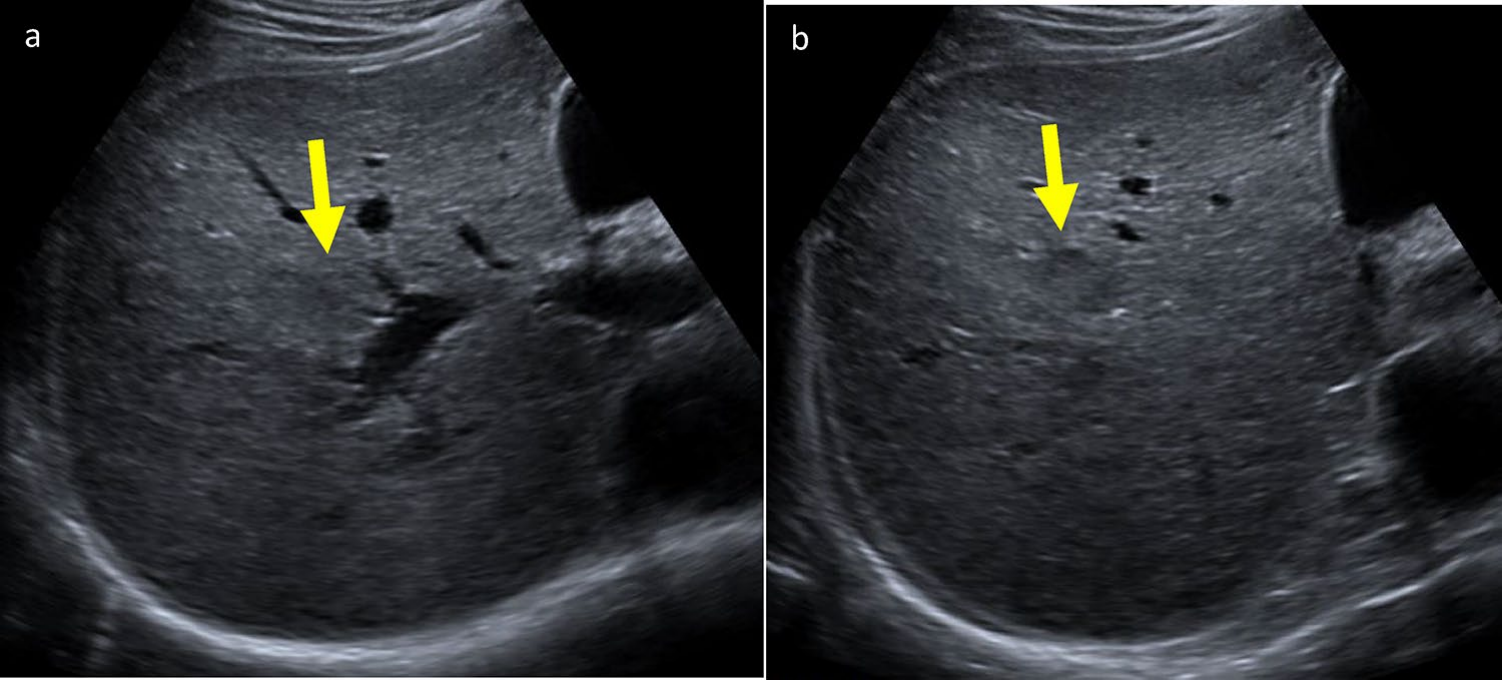
Cine sweeps clarify questionable finding on static grayscale images in a 63-year-old male with advanced fibrosis undergoing HCC surveillance. **a** Transverse grayscale ultrasound image shows a possible hypoechoic area (arrow) adjacent to right portal vein. **b** Select transverse grayscale image from a transverse right lobe cine sweep confirms a 2 cm solid hypoechoic nodule (arrow) that was categorized as LR-M on subsequent CEUS

Defining standard cine sweep acquisitions may decrease resistance to cine sweep acquisitions. The overwhelming majority of the LI-RADS US Surveillance working group members incorporate cine sweeps into their HCC surveillance protocols. Cine sweeps may improve liver visualization scores by allowing radiologists to find abnormalities that the sonographer may have overlooked, or conversely, dismiss an observation that may be due to an artifact. Overall, cine sweeps can help confirm or dismiss observations, improve visualization scores, provide a thorough assessment of the liver and focal lesions, allow retrospective review, and standardize exams Fig. 9.

### Challenge #7: implementing Surveillance US LI-RADS at your institution

Although US-based HCC surveillance is an integral part of the AASLD HCC Practice Guidance, [4] a recent survey of non-academic radiology practices predominantly from North America (69.4% USA, 25% Canada, 5.6% other) showed that less than 10% of practices consistently used the US LI-RADS algorithm for HCC surveillance [35]. Interestingly, in a separate international survey (22.5% Asia, 6% Europe, 19.9% North America, 26.5% South America, 25.2% Australia) showed any form of LI-RADS was used by 66% of respondents, and that ultrasound was the most common modality used for surveillance [36]. US LI-RADS was used by 17.2% of respondents whereas CT/MRI LI-RADS was used by 87.4% of respondents [36].

These surveys show that there is ample opportunity to improve utilization of LI-RADS US Surveillance, particularly in North American (non-academic) radiology practices. A barrier to the implementation of LI-RADS US Surveillance includes limited familiarity of radiologists with the algorithm.

Changes that may increase adoption of LI-RADS US Surveillance include the following. First, the new 2023 AASLD Practice Guidance has embraced reporting both the LI-RADS US Surveillance US Visualization Score as well as the US Category. Hepatologists familiar with the AASLD recommendations will therefore request usage of LI-RADS US Surveillance in assessment of patients at risk for HCC as this will inform their clinical decisions.

Second, the growing use of structured reporting enables the autopopulation of standardized templates, which will facilitate radiologists’ adoption of LI-RADS US Surveillance [37] Table 3. These templates should reference the ACR LI-RADS US Surveillance website to educate both the reporting radiologist and the referring clinician: https://www.acr.org/-/media/ACR/Files/RADS/LI-RADS/LI-RADS-US-Surveillance-v2024-Core.pdf

**Table 3:** Sample ACR LI-RADS US surveillance dictation template which can be adapted to your institution’s preferences

Sample Dictation Template
HISTORY: Etiology of chronic liver disease/cirrhosis if known COMPARISON: [] BIOMARKERS (optional): [] TECHNIQUE: Grayscale and color Doppler images of the abdomen were obtained FINDINGS: Liver: Visualization: [VIS-A, VIS-B, VIS-C]. (If VIS-B or C, state reason why) Liver morphology/parenchyma/contour: [] Liver observation(s): [] Liver vasculature: [portal vein and hepatic veins] Bile ducts: [] Gallbladder: [] Spleen: [size] Other organs (optional): [pancreas, aorta, IVC, kidneys] Ascites: [] Other findings: [Varices, paraumbilical vein, lymph nodes, etc.] IMPRESSION: 1. [Overall summary including liver and portal hypertension findings if present.] 2. LI-RADS US Category: [US-1 Negative. Repeat surveillance US in 6 months is recommended./US-2 Subthreshold observation. Observation detected that may warrant short-interval US surveillance in 3–6 months. If observation remains < 1 cm after 2 exams or is no longer seen, may recategorize as US-1 Negative/US-3 Positive. Further characterization with diagnostic contrast-enhanced imaging is recommended.] 3. LI-RADS US Visualization Score: [VIS-A—Limitations if any are unlikely to meaningfully affect exam sensitivity./VIS-B—Limitations may obscure small (< 10 mm) observations./C—Limitations significantly lower sensitivity for liver observations. Recommendation for VIS-C: Recommend repeat ultrasound surveillance exam within 3 months × 1 OR consider alternative surveillance strategy. Risk factors for VIS-C include MASH or EtOH cirrhosis, Child-Turcotte-Pugh (CTP) Class B or C cirrhosis, or BMI ≥ 35 kg/m^2^ https://www.acr.org/-/media/ACR/Files/RADS/LI-RADS/LI-RADS-US-Surveillance-v2024-Core.pdf Optional text: Referrer should review AFP values, determine if AFP is positive, and alter management recommendations accordingly

Finally, as in any new undertaking, local radiology and clinical champions who promote the surveillance program are critical in ensuring both successful adoption and ongoing high-quality implementation of LI-RADS US Surveillance.

## Conclusion

LI-RADS US Surveillance v2024 is a system that standardizes the technique, interpretation, and management of patients undergoing US for HCC surveillance. Many pitfalls can be overcome by improving visualization through optimizing technique and training sonographers and radiologists. In addition, the LI-RADS Visualization Score C identifies patients for whom US screening may have limited sensitivity and now has a recommendation to repeat an earlier US, or proceed to alternative screening modalities such as CT or MRI. Furthermore, in conjunction with US, CT, and MRI, biomarker panels will likely play an important future role in shaping a patient-centered approach to HCC surveillance [38]. Lastly, in the era of value based imaging and limited healthcare resources, an investment in improving US technique and interpretation could lead to higher US sensitivity, cost savings, improved patient compliance, and increased quality adjusted life years.

## Data Availability

No datasets were generated or analysed during the current study.
